# Congenital Hypertrophic Stenosis of the Pylorus

**Published:** 1907-04-27

**Authors:** 


### Points in Surgery.

### CONGENITAL HYPERTROPHIC STENOSIS OF THE PYLORUS.

Only during the last 10 years or so has this condition been commonly recognised, as previously the cases were not differentiated from those of ordinary infantile marasmus with vomiting. The characteristic symptom is forcible vomiting after food, commencing within the first month or two after birth, steadily increasing in severity, giving rise to extreme wasting, and finally, unless successfully treated, leading to death from inanition. In addition, gastric peristalsis is often readily seen and a hard tumour can usually be felt in the position of the pylorus. Signs of gastric catarrh commonly develop in the later stages and constipation becomes increasingly marked. Considerable uncertainty exists as to the real nature and cause of the disease, whether it is a true hypertrophy of the circular muscular fibres at the pyloric orifice, or whether this is merely a secondary condition due to increased irritability and activity (spasm) of the muscle, excited possibly by some alteration in the character of the gastric secretion.

It was, however, on the question of treatment that the chief interest of the discussion at the recent meeting of the Clinical Society centred, for on this all-important point marked divergencies of opinion presented themselves. Several of the speakers, both physicians and surgeons, maintained strongly that, in practically all the really serious cases, by far the best, if not the only chance, of complete recovery was afforded by a timely surgical operation, some form of pyloroplasty being preferable to gastroenterostomy. Certainly the figures brought forward by Dr. Cautley and Mr. Burghard strongly support this contention and contrast markedly with the very unfavourable results, obtained by some of the speakers, with purely medical treatment, especially with the statement of Dr. Ashby, of Manchester, that the eleven cases he had treated had all died. On the other hand, Dr. Still, and even more notably Dr. Hutchison, have had very good results without surgical intervention in cases which were apparently quite as severe as those subjected to operation. The essential feature of the medical treatment is regular washing-out of the stomach, at least once or twice a day at first, and later on every other day as the child begins to improve. At the same time, the diet must be carefully regulated, various combinations of albumen water, whey, peptonised milk, and humanised milk being given. Dieting alone, however, no matter how well carried out, is quite inadequate to effect much, if any, improvement, seeing that when the vomiting first begins the child is often being fed exclusively on the breast. But even if medical treatment is successful, it calls for much time and attention, as it must usually be continued for a long period. It is probably too soon to attempt to decide finally on the respective merits of these two different methods of treatment; but so far as our present knowledge goes the position may perhaps be summed up as follows: The first essential is early diagnosis of the disease, and this may be by no means easy, seeing that it has probably not yet obtained full recognition throughout the profession. It is so natural to attribute the vomiting to the ordinary gastric disturbance of infancy, especially as after a time some gastric catarrh is very prone to develop. The result is that one food after another is tried, the vomiting all the time getting worse and worse, until the infant is completely worn out, and either dies from some intercurrent disease or, perhaps, expires suddenly from sheer inanition. Whenever a young baby begins to vomit shortly after taking food and without obvious cause, and no improvement occurs when any apparent error in the diet has been corrected, the existence of this disease of the pylorus should be suspected, visible peristalsis should be carefully looked for, and the abdomen should be palpated day by day for a pyloric tumour. Once the diagnosis is clear, careful and systematic lavage should be resorted to, and the child's weight noted week by week. Rapid recovery is not to be expected, and more or less irregularity in the course of improvement is common. A gain of a very few ounces each week must be regarded as satisfactory. Should, however, the child continue steadily to lose ground, or should systematic lavage be for any reason impracticable, recourse should be had to surgery before the patient has become excessively emaciated, and, if possible, before gastric catarrh is firmly established.

